# Supplementary material for: Systematic microscopical analysis reveals obligate synergy between extracellular matrix components during Bacillus subtilis colony biofilm development
Source: Biofilm. 2022 Aug 24;4:100082. doi: 10.1016/j.bioflm.2022.100082 (PMC9486643; doi:10.1016/j.bioflm.2022.100082)
Supplement: Multimedia component 1 [file mmc1.docx]

# Supplemental Figures and Movie Legends


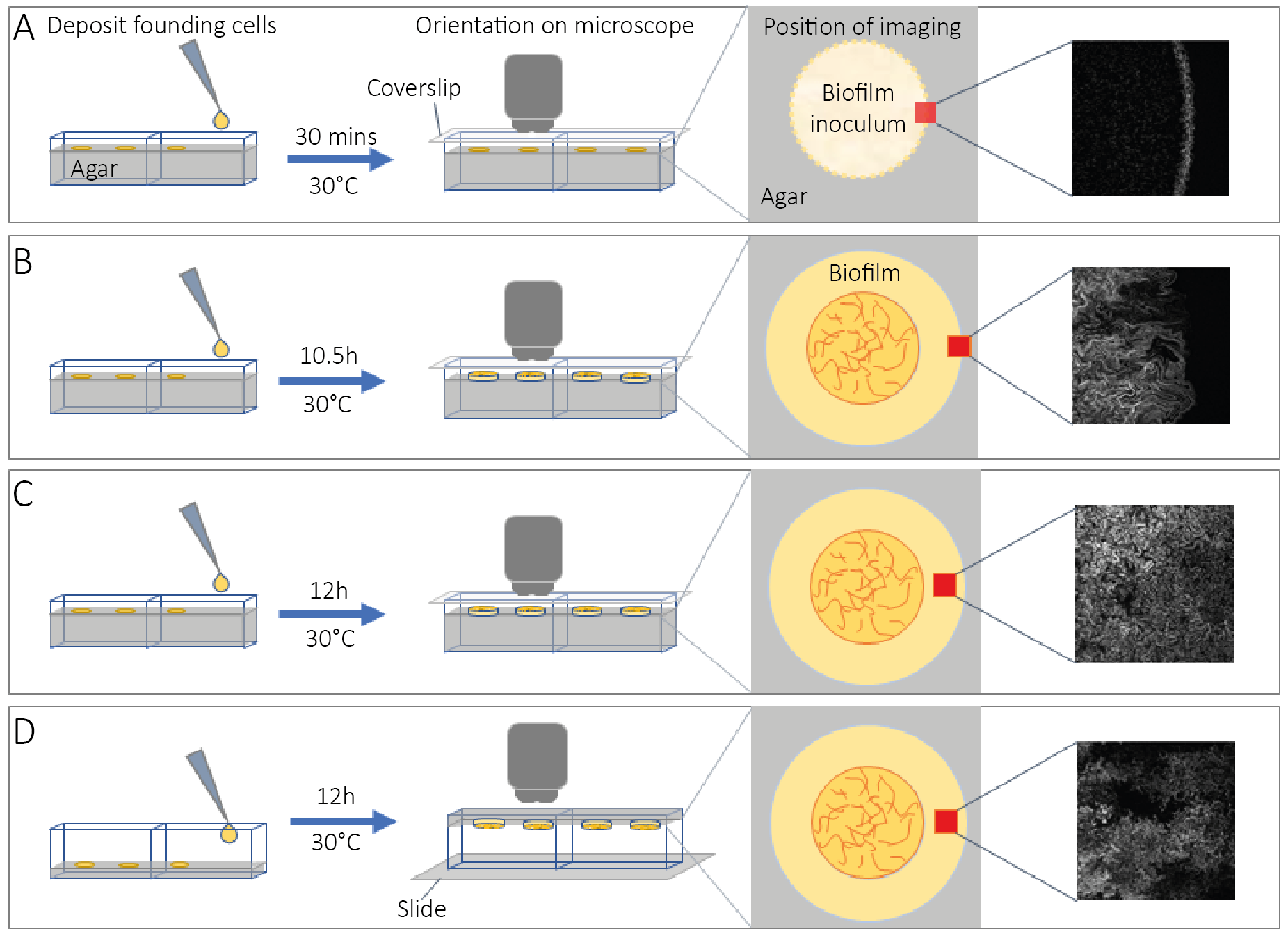


**Figure S 1 Schematic of imaging experiments** – Time-lapse confocal microscopy was performed on biofilms from different perspectives and at various points in development. **(A - C)** Lab-Tek II chambers were filled with MSgg agar (see Methods) to leave a small headspace for the biofilm to grow; **(D)** Lab-Tek II chambers were covered with a thin pad of MSgg agar to allow visualisation of the underside of the biofilm. In all cases, cells were deposited and the chambers were incubated before imaging for: 30 minutes (A); 10.5 hours (B); or 12 hours (C, D). Lab-Tek lids were removed and replaced with a long coverslip to protect the biofilm from warm airflow in the microscope chamber while allowing imaging of the biofilm-air interface (A-C) or inverted onto a microscope slide for imaging of the biofilm-agar interface (D). Fields of view were set to capture the edge of the biomass (A, B) or a region entirely within the biomass but near to the edge (C, D).


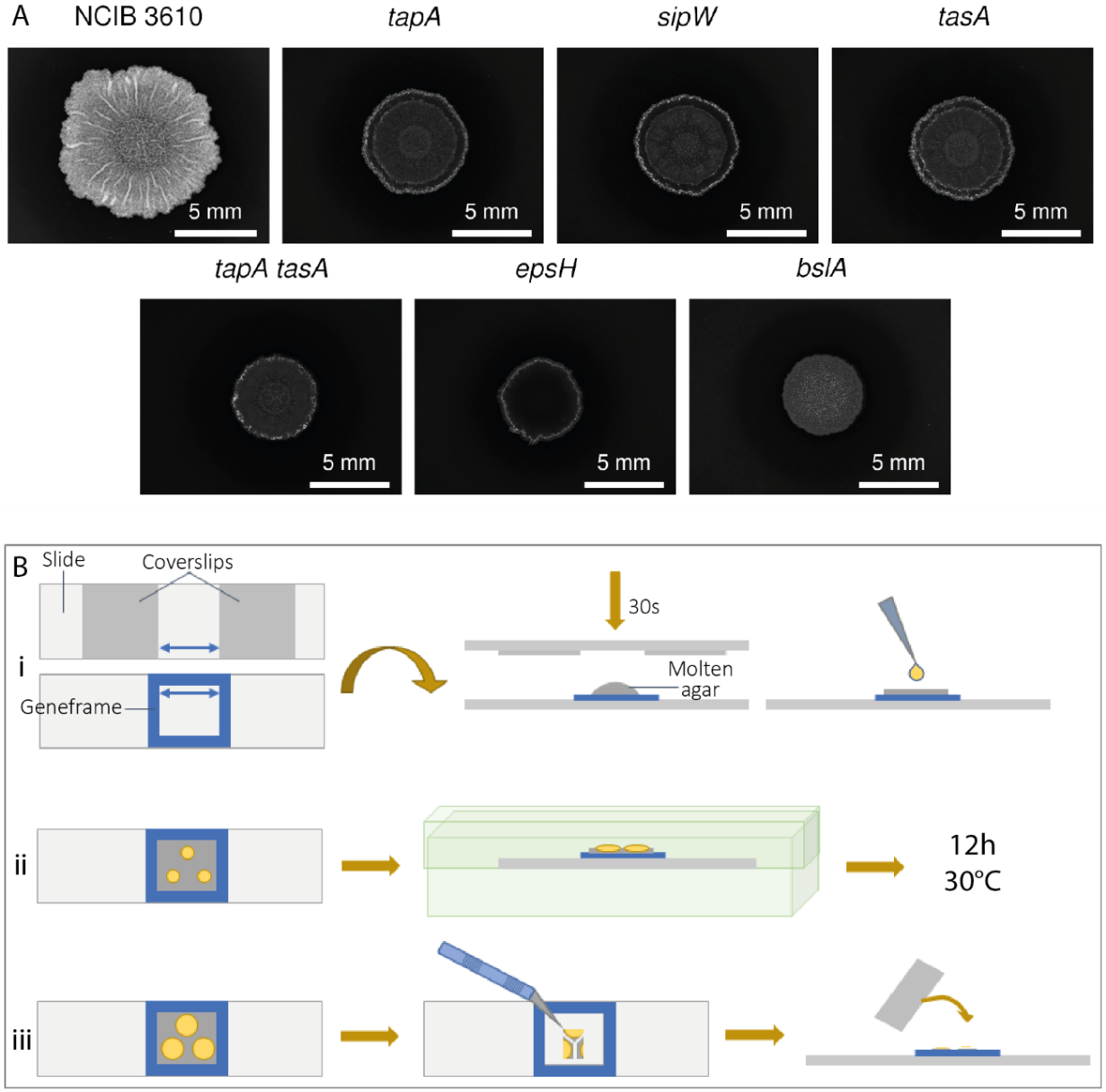


**Figure S 2**. **Experimental summary for strains expressing ftsZ-gfp**. **(A)** Wild type and matrix mutant strains producing the FtsZ-GFP fusion protein and the fluorophore mKate2 display morphologies akin to their parent strains. Strains shown are: NCIB 3610 background NRS6781; tapA NRS6793; sipW NRS6794; tasA NRS6795; tapA tasA NRS7525; epsH NRS7526; bslA NRS7527. The colony biofilms were grown at 30^o^C for 48 hours before imaging; **(B)** Experimental setup for high-resolution imaging of strains expressing ftsZ-gfp: (i) An adhesive Geneframe was attached to a glass side, and a flat mould was prepared by affixing two coverslips to a second slide, spaced apart to match the width of the aperture of the Geneframe (blue arrows). Molten MSgg agar was pipetted into the Geneframe and immediately flattened with the mould, held down for 30 seconds to set. Cells were deposited onto the agar pad, which is slightly raised relative to the edge of the Geneframe to allow for shrinkage during incubation. (ii) After inoculating three biofilms onto the agar, the slide was placed into a humidified box (microtube storage box with water in the outer positions) and incubated at 30°C for 12 hours. (iii) Immediately before imaging, the slide was removed from the box and most of the agar and unnecessary biomass was cut away with a scalpel to increase air space and reduce the oxygen demand. A gas-permeable coverslip was applied to complete the mounting of the sample.


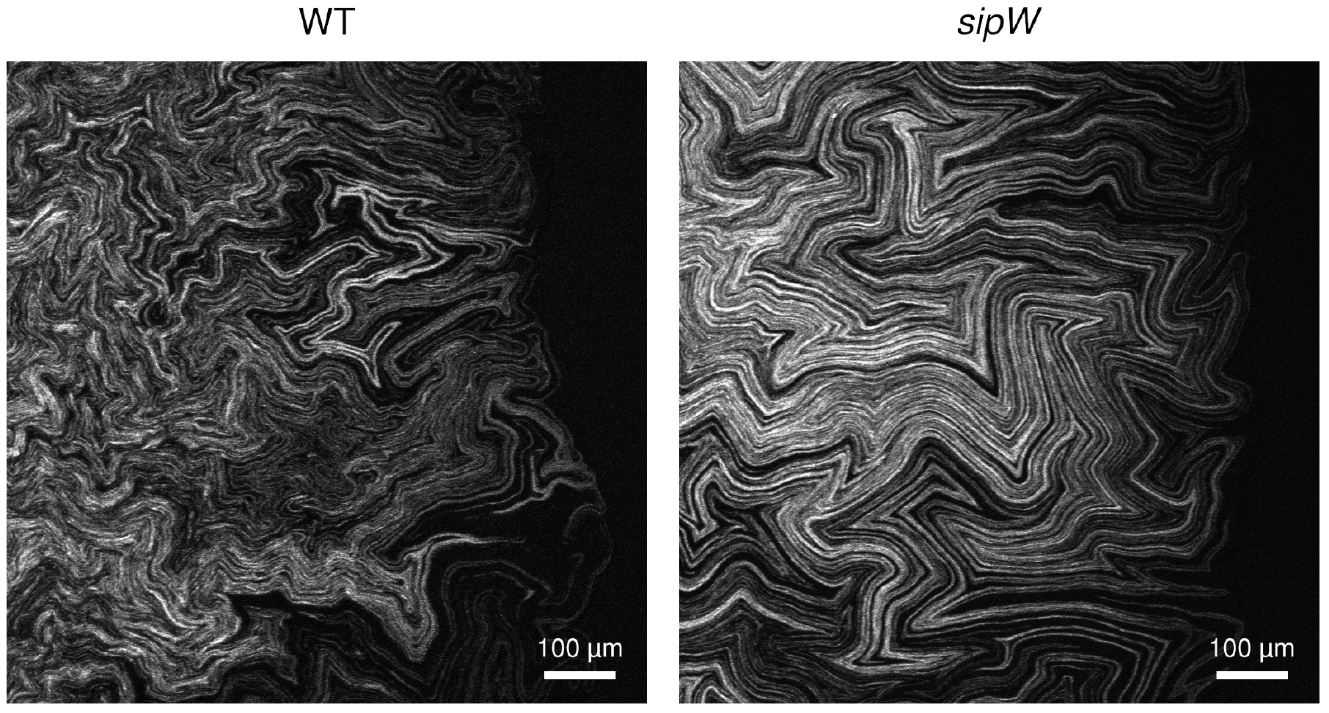


**Figure S 3 Aerial structure formation at the colony biofilm periphery**. Confocal imaging of NCIB 3610 colony biofilm edges shows the development of complex structures within the width of a field of view, which contrasts with the maintenance of monolayered chains in a sipW colony biofilm. Still images are taken from the time-lapse microscopy of Movie 2 and Movie S2 B respectively. Colony biofilms were formed upon coculture of NCIB 3610 with NRS1473 (WT) and NRS5488 with NRS6718 (sipW). The images shown are from approximately 14 hours after cell deposition.


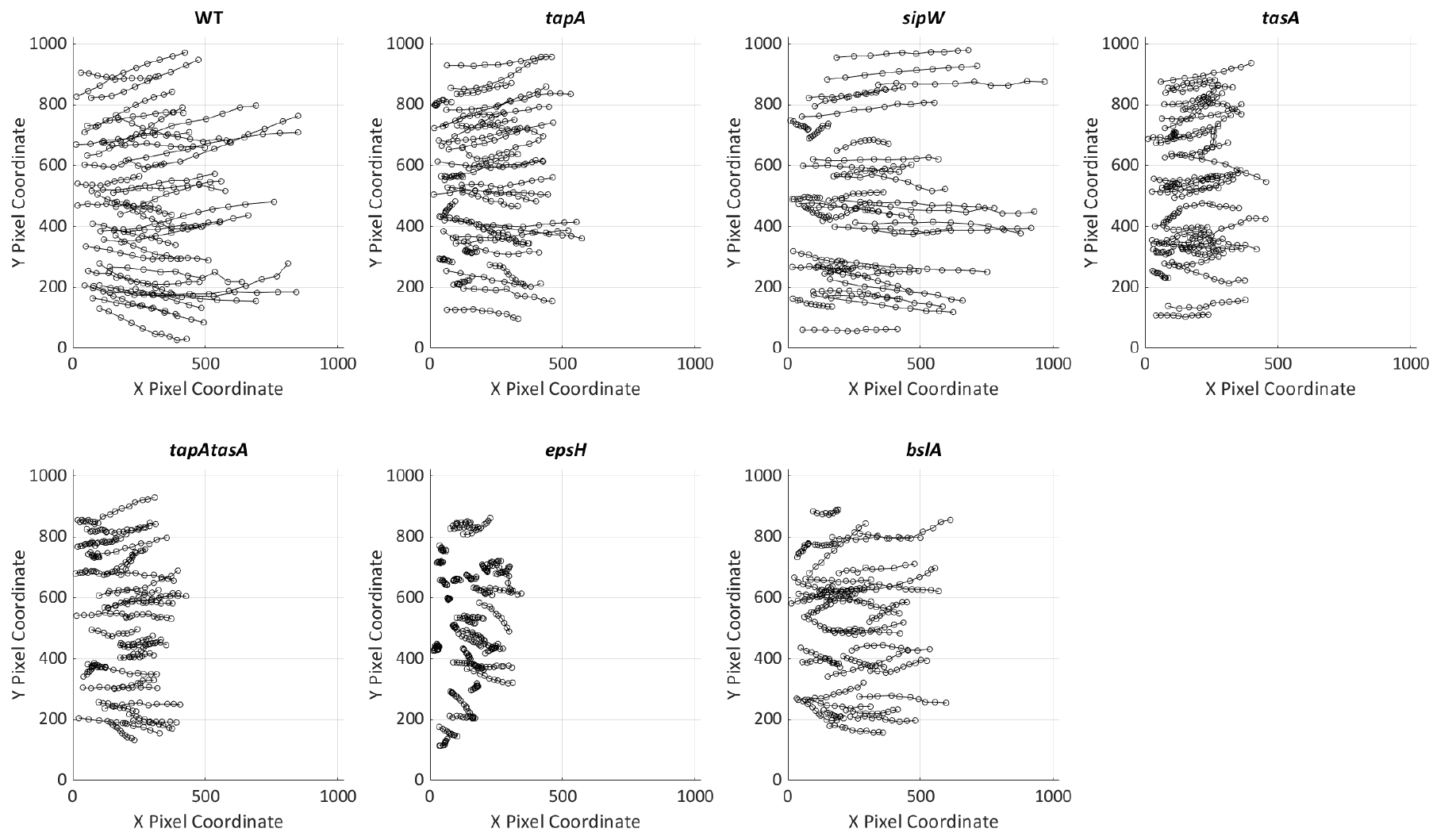


**Figure S 4 Tracking biofilm landmark movement over time**. Visibly recognisable landmarks had their locations specified by the placement of a region of interest (ROI) on each time-point in time-lapse imaging (see Fig Movie S3). Tracks are represented by a temporal projection of the coordinates of the ROIs on the 1024 x 1024-pixel image space. In total, 6 tracks from each of 6 time-lapse images are shown per genotype.


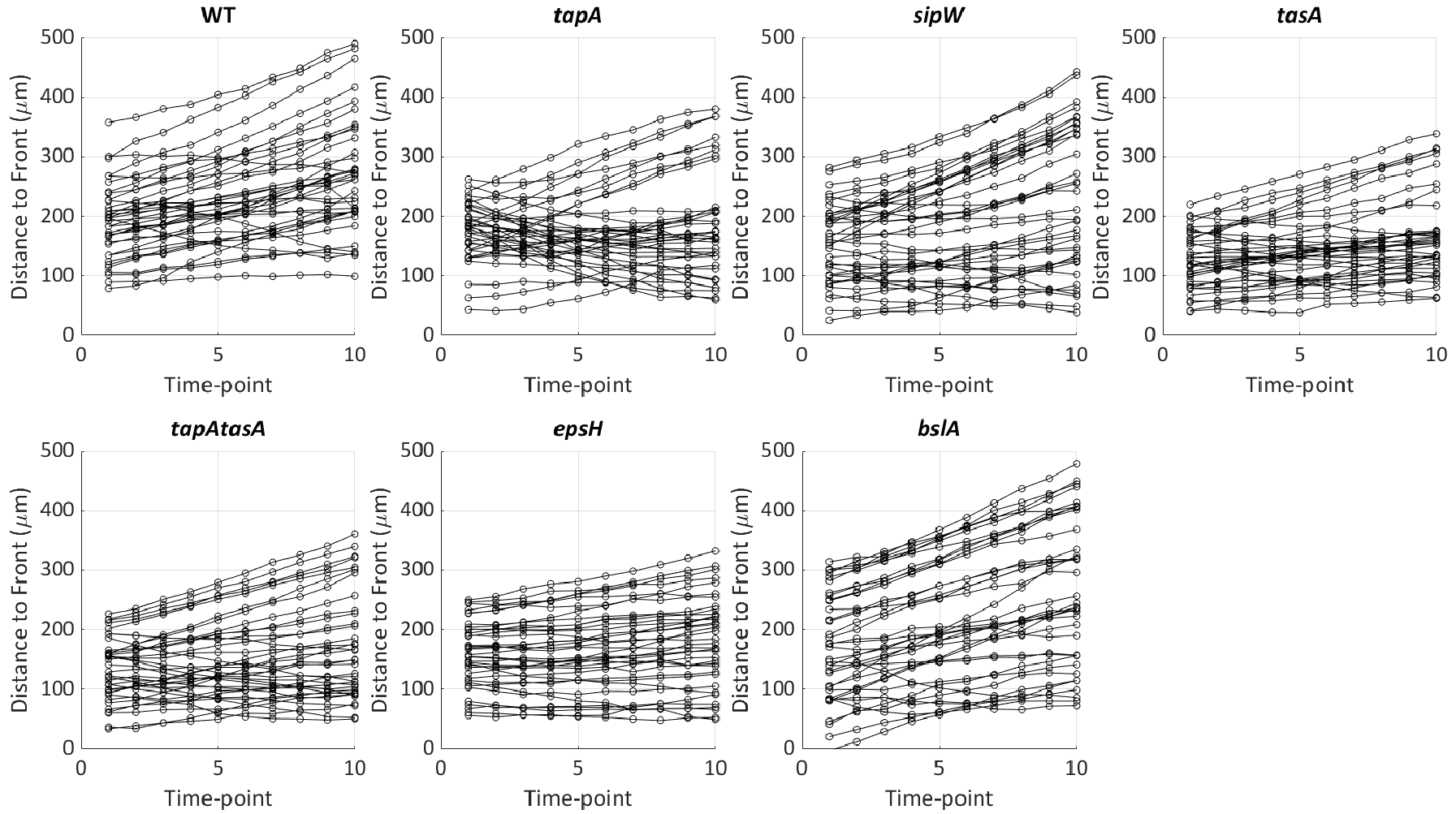


**Figure S 5 Landmark motion relative to the edge of the biofilm**. For each landmark tracked across time-lapse images, the distance to the expanding edge (Distance to Front) of the biofilm was measured at each time-point (see Movie S3, ‘d’). In the plots, approximately horizontal lines show landmarks keeping pace with the expanding edge, whereas positive or negative gradient lines show landmarks falling behind or catching up with the edge, respectively


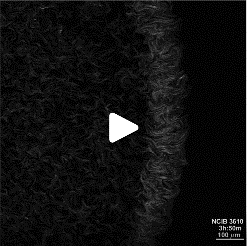


**Movie 1 Initial cell growth.** Time-lapse confocal microscopy of strains NCIB 3610 (80% population) and GFP-producing NCIB 3610 derivative NRS1473 (20% population) cocultured from 30 minutes after cell deposition. Z-stacks were acquired every 10 minutes for 10 hours, and a maximum intensity projection is presented. The timestamp is the approximate age of the biofilm since cells were deposited and the scale bar is 100 µm.


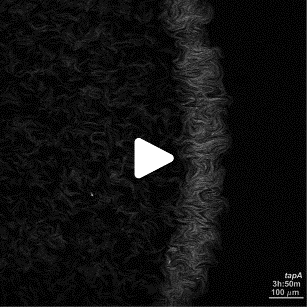


**Movie 2 Initial tapA cell growth**. Time-lapse confocal microscopy of cocultured strains of the genotypes indicated below contains 20% GFP-producing cells at the point of inoculation from 30 minutes after cell deposition. Z-stacks were acquired every 10 minutes for 10 hours, and a maximum intensity projection is presented. The timestamp is the approximate age of the biofilm since cells were deposited and the scale bar is 100 µm. The strains cocultured were tapA NRS3936/NRS6723.


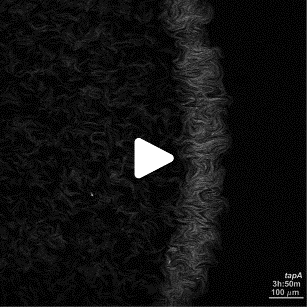


**Movie 3 Initial sipW cell growth**. Time-lapse confocal microscopy of cocultured strains of the genotypes indicated below contains 20% GFP-producing cells at the point of inoculation from 30 minutes after cell deposition. Z-stacks were acquired every 10 minutes for 10 hours, and a maximum intensity projection is presented. The timestamp is the approximate age of the biofilm since cells were deposited and the scale bar is 100 µm. The strains cocultured were sipW NRS5488/NRS6718.


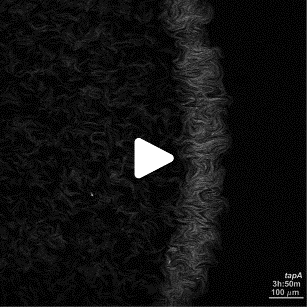


**Movie 4 Initial tasA cell growth**. Time-lapse confocal microscopy of cocultured strains of the genotypes indicated below contains 20% GFP-producing cells at the point of inoculation from 30 minutes after cell deposition. Z-stacks were acquired every 10 minutes for 10 hours, and a maximum intensity projection is presented. The timestamp is the approximate age of the biofilm since cells were deposited and the scale bar is 100 µm. The strains cocultured were tasA NRS5267/NRS6724.


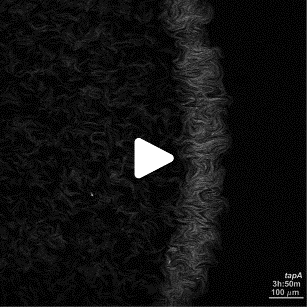


**Movie 5 Initial tapA tasA cell growth**. Time-lapse confocal microscopy of cocultured strains of the genotypes indicated below contains 20% GFP-producing cells at the point of inoculation from 30 minutes after cell deposition. Z-stacks were acquired every 10 minutes for 10 hours, and a maximum intensity projection is presented. The timestamp is the approximate age of the biofilm since cells were deposited and the scale bar is 100 µm. The strains cocultured were tapA tasA NRS5748/NRS6727.


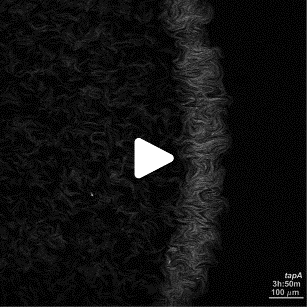


**Movie 6 Initial epsH cell growth**. Time-lapse confocal microscopy of cocultured strains of the genotypes indicated below containing 20% GFP-producing cells at the point of inoculation from 30 minutes after cell deposition. Z-stacks were acquired every 10 minutes for 10 hours, and a maximum intensity projection is presented. The timestamp is the approximate age of the biofilm since cells were deposited and the scale bar is 100 µm. The strains cocultured were epsH NRS5906/NRS6728.


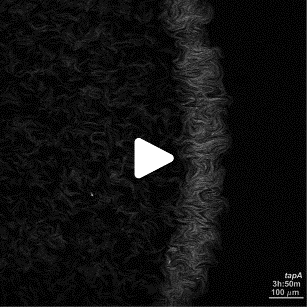


**Movie 7 Initial bslA cell growth**. Time-lapse confocal microscopy of cocultured strains of the genotypes indicated below containing 20% GFP-producing cells at the point of inoculation from 30 minutes after cell deposition. Z-stacks were acquired every 10 minutes for 10 hours, and a maximum intensity projection is presented. The timestamp is the approximate age of the biofilm since cells were deposited and the scale bar is 100 µm. The strains cocultured were bslA NRS2097/NRS5131.


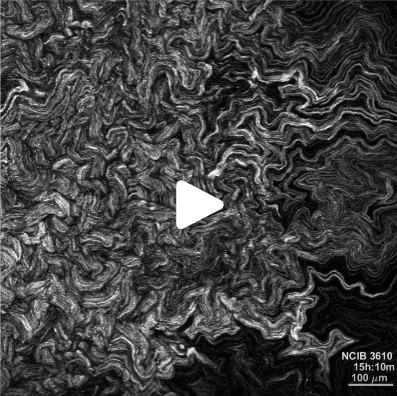


**Movie 8** **Cell chains emanate from the NCIB 3610 biofilm periphery**. Time-lapse confocal microscopy of strains NCIB 3610 (80% population) and the NCIB 3610 GFP-producing derivative NRS1473 (20% population) from 10.5 hours after cell deposition and incubation at 30°C. Z-stacks were acquired every 10 minutes for 14 hours, and a maximum intensity projection is presented. The timestamp is the approximate age of the biofilm since cells were deposited and the scale bar is 100 µm.


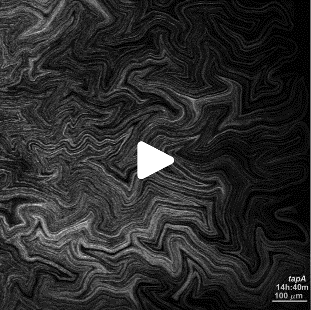


**Movie 9 Cell chains emanate from the tapA biofilm periphery.** Time-lapse confocal microscopy of cocultured strains of the genotypes indicated below containing 20% GFP-producing cells at the point of inoculation from 10.5 hours after cell deposition and incubation at 30°C. Z-stacks were acquired every 10 minutes for 14 hours, and a maximum intensity projection is presented. The timestamp is the approximate age of the biofilm since cells were deposited and the scale bar is 100 µm. The strains cocultured were tapA NRS3936/NRS6723.


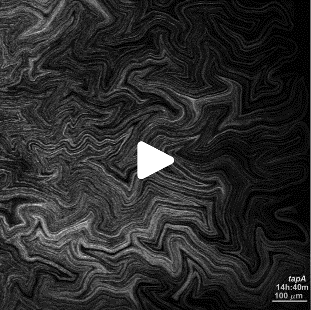


**Movie 10 Cell chains emanate from the sipW biofilm periphery.** Time-lapse confocal microscopy of cocultured strains of the genotypes indicated below containing 20% GFP-producing cells at the point of inoculation from 10.5 hours after cell deposition and incubation at 30°C. Z-stacks were acquired every 10 minutes for 14 hours, and a maximum intensity projection is presented. The timestamp is the approximate age of the biofilm since cells were deposited and the scale bar is 100 µm. The strains cocultured were sipW NRS5488/NRS6718.


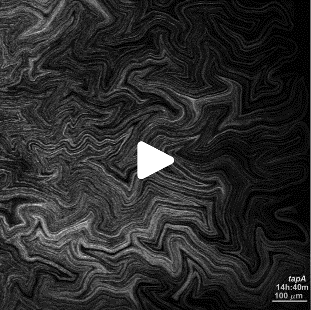


**Movie 11 Cell chains emanate from the tasA biofilm periphery.** Time-lapse confocal microscopy of cocultured strains of the genotypes indicated below containing 20% GFP-producing cells at the point of inoculation from 10.5 hours after cell deposition and incubation at 30°C. Z-stacks were acquired every 10 minutes for 14 hours, and a maximum intensity projection is presented. The timestamp is the approximate age of the biofilm since cells were deposited and the scale bar is 100 µm. The strains cocultured were tasA NRS5267/NRS6724.


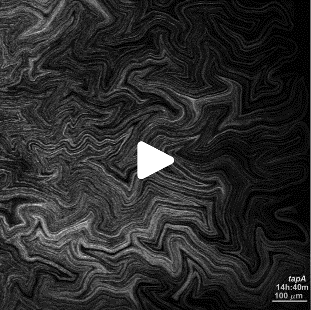


**Movie 12 Cell chains emanate from the tapA tasA biofilm periphery.** Time-lapse confocal microscopy of cocultured strains of the genotypes indicated below containing 20% GFP-producing cells at the point of inoculation from 10.5 hours after cell deposition and incubation at 30°C. Z-stacks were acquired every 10 minutes for 14 hours, and a maximum intensity projection is presented. The timestamp is the approximate age of the biofilm since cells were deposited and the scale bar is 100 µm. The strains cocultured were tapA tasA NRS5748/NRS6727.


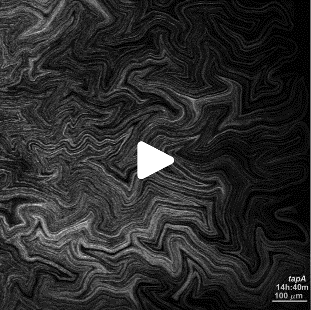


**Movie 13 Cell chains emanate from the epsH biofilm periphery.** Time-lapse confocal microscopy of cocultured strains of the genotypes indicated below containing 20% GFP-producing cells at the point of inoculation from 10.5 hours after cell deposition and incubation at 30°C. Z-stacks were acquired every 10 minutes for 14 hours, and a maximum intensity projection is presented. The timestamp is the approximate age of the biofilm since cells were deposited and the scale bar is 100 µm. The strains cocultured were epsH NRS5906/NRS6728.


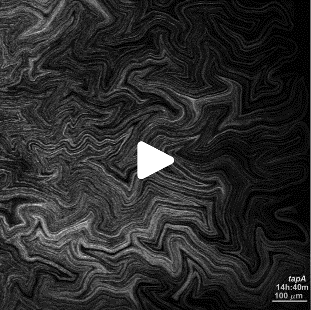


**Movie 14 Cell chains emanate from the bslA biofilm periphery.** Time-lapse confocal microscopy of cocultured strains of the genotypes indicated below containing 20% GFP-producing cells at the point of inoculation from 10.5 hours after cell deposition and incubation at 30°C. Z-stacks were acquired every 10 minutes for 14 hours, and a maximum intensity projection is presented. The timestamp is the approximate age of the biofilm since cells were deposited and the scale bar is 100 µm. The strains cocultured were bslA NRS2097/NRS5131.


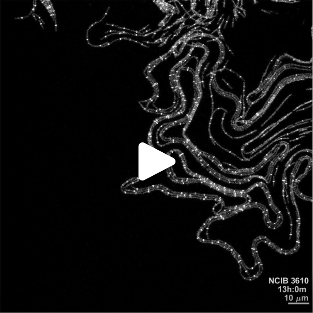


**Movie 15 Example of single cell division time within the context of a colony biofilm.** Time-lapse confocal microscopy of mixed strains NCIB 3610 (80%) and FtsZ-GFP-producing NRS6781 (20%) from 12 hours after cell deposition and incubation at 30°C. Z-stacks were acquired every 10 minutes, and a maximum intensity projection is presented. The timestamp is the approximate age of the biofilm since cells were deposited and the scale bar is 10 µm


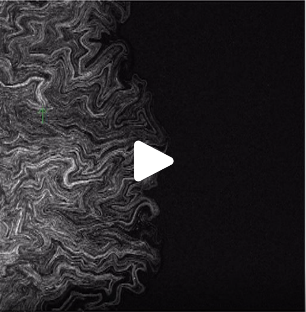


**Movie 16 Example of biomass landmark motion**. Time-lapse confocal microscopy of mixed strains NCIB 3610 (80%) and GFP-producing NRS1473 (20%) from 10.5 hours after cell deposition and incubation at 30°C. Z-stacks were acquired every 10 minutes for 14 hours, and a maximum intensity projection is presented. Green arrows highlight aerial structures that form a short distance from the expanding edge of the biofilm.


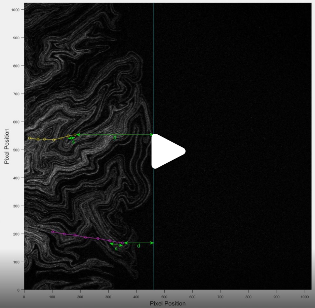


**Movie 17 Tracking and measuring landmark motion**. Time-lapse confocal microscopy of cocultured strains NCIB 3610 (80%) and GFP-producing NRS1473 (20%) from 10.5 hours after cell deposition and incubation at 30°C. Z-stacks were acquired every 10 minutes for 14 hours, and a maximum intensity projection is presented. In this example, two visibly recognisable landmarks have their locations defined by the placement of an ROI at each time-point, represented by the emerging yellow and magenta tracks. Two measurements are then made: step-size ‘s’ is the Euclidean distance a landmark travelled between discrete time points; measurement ‘d’ is the distance from the landmark to the expanding edge of the biofilm.


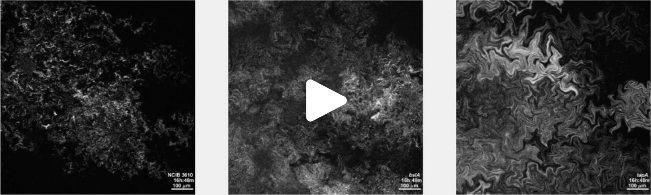


**Movie 18 Biomass structure development at the biofilm-agar interface**. Time-lapse confocal microscopy at the biofilm-agar interface of mixed strains of the genotypes stated containing 20% GFP-producing cells. Imaging was begun at 12 hours after cell deposition and incubation at 30°C. Z-stacks were acquired every 18 minutes, and a maximum intensity projection is presented. The timestamp is the approximate age of the biofilm since cells were deposited and the scale bar is 100 µm. Strains used are NCIB 3610/NRS1473, bslA NRS2097/NRS5131, and tasA NRS5267/NRS6724.


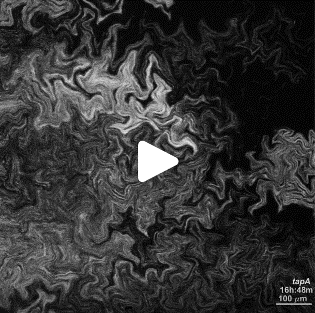


**Movie 19 Biomass structure development at the tapA** **biofilm-agar interface**. Time-lapse confocal microscopy at the biofilm-agar interface of mixed strains of the genotypes stated containing 20% GFP-producing cells at the point of inoculation. Imaging started at 12 hours after cell deposition and incubation was at 30°C. Z-stacks were acquired every 18 minutes, and a maximum intensity projection is presented. The timestamp is the approximate age of the biofilm since cells were deposited and the scale bar is 100 µm. The strains cocultured were tapA NRS3936/NRS6723.


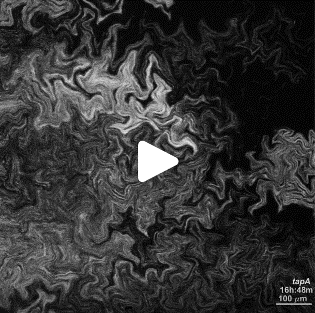


**Movie 20 Biomass structure development at the sipW biofilm-agar interface**. Time-lapse confocal microscopy at the biofilm-agar interface of mixed strains of the genotypes stated containing 20% GFP-producing cells at the point of inoculation. Imaging started at 12 hours after cell deposition and incubation was at 30°C. Z-stacks were acquired every 18 minutes, and a maximum intensity projection is presented. The timestamp is the approximate age of the biofilm since cells were deposited and the scale bar is 100 µm. The strains cocultured were sipW NRS5488/NRS6718.


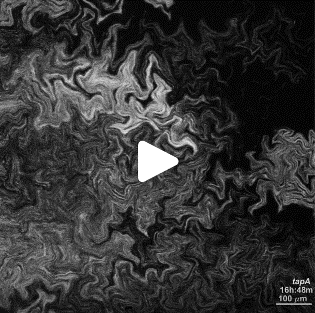


**Movie 21 Biomass structure development at the tasA** **biofilm-agar interface**. Time-lapse confocal microscopy at the biofilm-agar interface of mixed strains of the genotypes stated containing 20% GFP-producing cells at the point of inoculation. Imaging started at 12 hours after cell deposition and incubation was at 30°C. Z-stacks were acquired every 18 minutes, and a maximum intensity projection is presented. The timestamp is the approximate age of the biofilm since cells were deposited and the scale bar is 100 µm. The strains cocultured were tasA NRS5267/NRS6724.


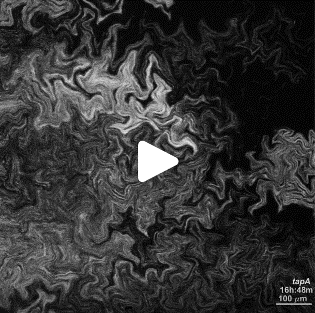


**Movie 22 Biomass structure development at the tapA tasA** **biofilm-agar interface**. Time-lapse confocal microscopy at the biofilm-agar interface of mixed strains of the genotypes stated containing 20% GFP-producing cells at the point of inoculation. Imaging started at 12 hours after cell deposition and incubation was at 30°C. Z-stacks were acquired every 18 minutes, and a maximum intensity projection is presented. The timestamp is the approximate age of the biofilm since cells were deposited and the scale bar is 100 µm. The strains cocultured were tapA tasA NRS5748/NRS6727.


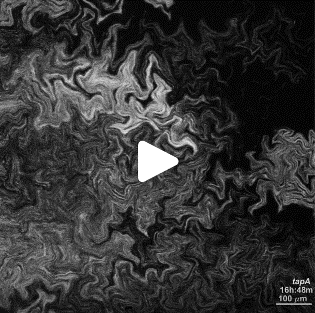


**Movie 23 Biomass structure development at the epsH** **biofilm-agar interface**. Time-lapse confocal microscopy at the biofilm-agar interface of mixed strains of the genotypes stated containing 20% GFP-producing cells at the point of inoculation. Imaging started at 12 hours after cell deposition and incubation was at 30°C. Z-stacks were acquired every 18 minutes, and a maximum intensity projection is presented. The timestamp is the approximate age of the biofilm since cells were deposited and the scale bar is 100 µm. The strains cocultured were epsH NRS5906/NRS6728.


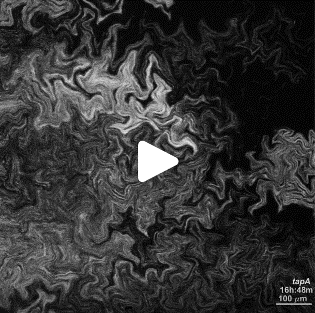


**Movie 24 Biomass structure development at the bslA** **biofilm-agar interface**. Time-lapse confocal microscopy at the biofilm-agar interface of mixed strains of the genotypes stated containing 20% GFP-producing cells at the point of inoculation. Imaging started at 12 hours after cell deposition and incubation was at 30°C. Z-stacks were acquired every 18 minutes, and a maximum intensity projection is presented. The timestamp is the approximate age of the biofilm since cells were deposited and the scale bar is 100 µm. The strains cocultured were bslA NRS2097/NRS5131.


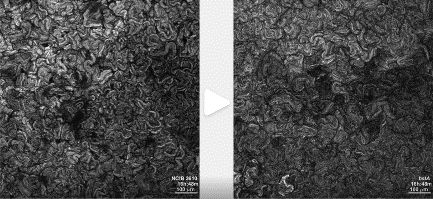


**Movie 25 Biomass movement at the biofilm-air interface.** Time-lapse confocal microscopy at the biofilm-air interface of mixed strains of the genotypes stated to contain 20% GFP-producing cells. Imaging was begun at 12 hours after cell deposition and incubation at 30°C. Z-stacks were acquired every 18 minutes, and a maximum intensity projection is presented. The timestamp is the approximate age of the biofilm since cells were deposited and the scale bar is 100 µm. Strains used are NCIB 3610/NRS1473, bslA NRS2097/NRS5131


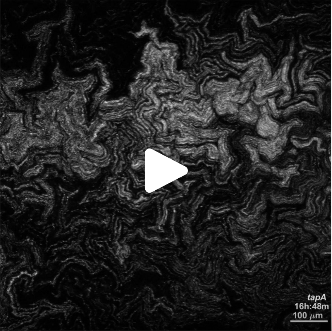


**Movie 26 Biomass movement at the** tapA **biofilm-air interface.** Time-lapse confocal microscopy at the biofilm-air interface of mixed strains of the genotypes stated containing 20% GFP-producing cells at the point of inoculation. Imaging started at 12 hours after cell deposition and incubation at 30°C. Z-stacks were acquired every 18 minutes, and a maximum intensity projection is presented. The timestamp is the approximate age of the biofilm since cells were deposited and the scale bar is 100 µm. The strains cocultured were tapA NRS3936/NRS6723.


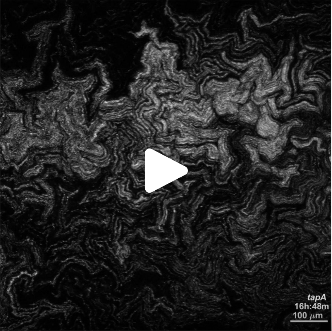


**Movie 27 Biomass movement at the** sipW **biofilm-air interface.** Time-lapse confocal microscopy at the biofilm-air interface of mixed strains of the genotypes stated containing 20% GFP-producing cells at the point of inoculation. Imaging started at 12 hours after cell deposition and incubation at 30°C. Z-stacks were acquired every 18 minutes, and a maximum intensity projection is presented. The timestamp is the approximate age of the biofilm since cells were deposited and the scale bar is 100 µm. The strains cocultured were sipW NRS5488/NRS6718.


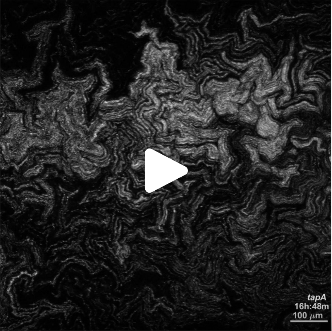


**Movie 28 Biomass movement at the tasA biofilm-air interface.** Time-lapse confocal microscopy at the biofilm-air interface of mixed strains of the genotypes stated containing 20% GFP-producing cells at the point of inoculation. Imaging started at 12 hours after cell deposition and incubation at 30°C. Z-stacks were acquired every 18 minutes, and a maximum intensity projection is presented. The timestamp is the approximate age of the biofilm since cells were deposited and the scale bar is 100 µm. The strains cocultured were tasA NRS5267/NRS6724.


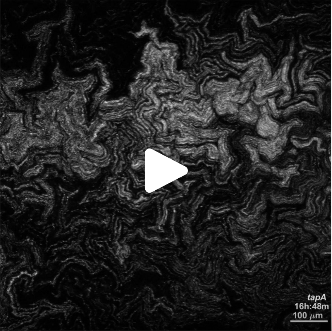


**Movie 29 Biomass movement at the tapA tasA biofilm-air interface.** Time-lapse confocal microscopy at the biofilm-air interface of mixed strains of the genotypes stated containing 20% GFP-producing cells at the point of inoculation. Imaging started at 12 hours after cell deposition and incubation at 30°C. Z-stacks were acquired every 18 minutes, and a maximum intensity projection is presented. The timestamp is the approximate age of the biofilm since cells were deposited and the scale bar is 100 µm. The strains cocultured were tapA tasA NRS5748/NRS6727.


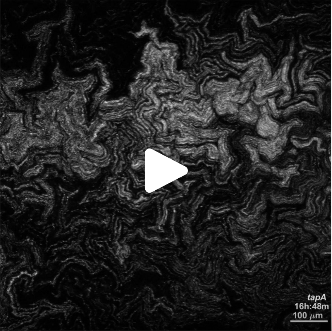


**Movie 30 Biomass movement at the epsH biofilm-air interface.** Time-lapse confocal microscopy at the biofilm-air interface of mixed strains of the genotypes stated containing 20% GFP-producing cells at the point of inoculation. Imaging started at 12 hours after cell deposition and incubation at 30°C. Z-stacks were acquired every 18 minutes, and a maximum intensity projection is presented. The timestamp is the approximate age of the biofilm since cells were deposited and the scale bar is 100 µm. The strains cocultured were epsH NRS5906/NRS6728.


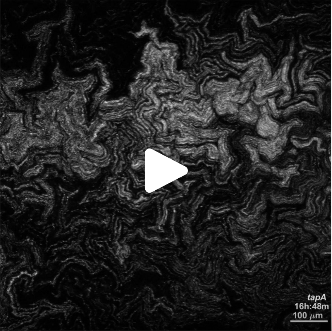


**Movie 31 Biomass movement at the bslA biofilm-air interface.** Time-lapse confocal microscopy at the biofilm-air interface of mixed strains of the genotypes stated containing 20% GFP-producing cells at the point of inoculation. Imaging started at 12 hours after cell deposition and incubation at 30°C. Z-stacks were acquired every 18 minutes, and a maximum intensity projection is presented. The timestamp is the approximate age of the biofilm since cells were deposited and the scale bar is 100 µm. The strains cocultured were bslA NRS2097/NRS5131).
